# Sentinel surveillance of acute febrile illness in a Brazilian Amazon region: laboratory findings and epidemiological implications

**DOI:** 10.1590/S1678-9946202668067

**Published:** 2026-10-02

**Authors:** Erick Matheus Garcia Barbosa, Antonio Charlys da Costa, Noely Evangelista Ferreira, Heuder Gustavo Oliveira Paião, Wilton Santos Freire, Anderson Vicente de Paula, Layla Honorato, Vanessa dos Santos Morais, Márlisson Octávio da Silva Rego, Edcelha Soares D’Athaide Ribeiro, Diuliana dos Santos Mendes, Júlia Pantoja Marques, Geovani de Oliveira Ribeiro, Steven Sol Witkin, Tania Regina Tozetto-Mendoza, Maria Cassia Mendes-Correa

**Affiliations:** 1 Universidade de São Paulo, Faculdade de Medicina, Departamento de Infectologia e Medicina Tropical, São Paulo, São Paulo, Brazil; 2 Universidade de São Paulo, Faculdade de Medicina, Instituto de Medicina Tropical de São Paulo, Laboratório de Investigação Médica (LIM-52), São Paulo, São Paulo, Brazil; 3 Superintendência de Vigilância em Saúde do Amapá, Laboratório de Saúde Pública do Amapá, Macapá, Amapá, Brazil; 4 Ministério da Saúde, Secretaria de Vigilância em Saúde e Ambiente, Coordenação-Geral de Laboratórios de Saúde Pública, Brasília, DF, Brazil; 5 Weill Cornell Medicine, Department of Obstetrics and Gynecology, New York, New York, USA

**Keywords:** Dengue, Leptospirosis, Arbovirus infections, Chikungunya fever, Parvovirus B19

## Abstract

The Amapa State, Brazil, in the Amazon region and bordering French Guiana, is a strategic sentinel location for infectious disease surveillance. Ecological pressures such as deforestation, artisanal gold mining, and high human mobility contribute to complex transmission dynamics of arboviruses, bacterial infections, and other pathogens. This study aims to characterize the etiologies of acute febrile illness (AFI) reported in Amapa State in 2018-2020, focusing on arbovirus circulation, bacterial contributions, and emerging pathogens. A retrospective analysis was carried out with 727 AFI cases. Laboratory diagnoses were established using molecular and serological assays, with systematic testing for different pathogens. Definitive diagnoses were obtained in 185 individuals (25.4%), yielding 209 positive results and including 23 cases of co-infection. Dengue virus was the predominant etiological agent (n=109, 52%), followed by leptospirosis (n=20, 9.5%), chikungunya virus (n=16, 7.6%), parvovirus B19 (n=16, 7.6%), measles (n=15, 7.1%), zika virus (n=10, 4.8%), mayaro virus (n=1,0.5%), and other bacterial infections (n=5, 2.4%). Isolated cases of hepatitis A and B, malaria, SARS-CoV-2, enterovirus, and acute Chagas disease were also identified. No hepatitis E virus or oropouche cases were detected. Dengue virus was the leading cause of AFI. Leptospirosis, chikungunya virus, and parvovirus B19 were other prevalent and often underrecognized contributors. The detection of additional viruses highlights Amapa State as a potential zone of “silent expansion” for emerging arboviruses. Co-detection of multiple pathogens underscores diagnostic complexity in regions with overlapping transmission. Strengthening surveillance, expanding laboratory capacity, and prioritizing differential diagnostics are essential to capture epidemiological dynamics and guide public health interventions.

## INTRODUCTION

Managing the prevention, diagnosis, and treatment of community-acquired acute febrile illness (AFI) constitutes a major public health challenge in Latin America due to its complex epidemiological profile and potentially profound social consequences^1^. A comprehensive understanding of their etiologies is essential for advancing clinical practice, guiding public health strategies, and informing policy development.

The ecology of the Amazon basin—characterized by deforestation, flooding, and high vector diversity—creates favorable conditions for arboviruses and bacterial pathogens, amplifying the risk of febrile outbreaks^2^. Thus, the region experiences a wide spectrum of febrile illnesses caused by viral, bacterial, and parasitic agents, reflecting diverse ecological and climatic conditions^3^.

The overlapping clinical manifestations of diseases such as dengue, malaria, leptospirosis, and rickettsial infections complicate accurate diagnosis. This diagnostic uncertainty often results in delayed or inappropriate treatment. Misdiagnosis may postpone life-saving antibiotic therapy, whereas indiscriminate prescribing contributes to antimicrobial resistance. The Pan American Health Organization^4^ has emphasized antimicrobial resistance as a regional priority, highlighting its impact on healthcare costs and mortality.

Community-acquired AFI contributes substantially to regional morbidity and mortality, considerably straining healthcare systems and requiring significant resource allocation for surveillance and management. Socioeconomic factors, including poverty and limited access to healthcare, exacerbate transmission dynamics and hinder effective disease control^5^.

Surveillance represents a critical component of disease control. The Brazilian Amazon is recognized as a global hotspot for emerging viruses^2^. Integrated screening systems may serve as sentinel mechanisms for early detection. As deforestation and mining activities intensify, human populations increasingly encounter novel vectors such as mosquitoes and midges, underscoring the importance of screening febrile patients for malaria and arboviruses6. Without routine screening, outbreaks may remain undetected, manifesting themselves only as “unexplained fevers.”

Amapa State, in the Eastern Brazilian Amazon, exemplifies one of the most complex diagnostic environments in the world. While dengue remains a major concern, the unique geography of the state—bordering French Guiana and the Atlantic Ocean, and dominated by dense rainforest and wetlands—heightens the risk of diverse infectious diseases. High rainfall and humidity render leptospirosis endemic, particularly in peri-urban areas such as Macapa and Santana^5^. Recent land-use transformations, including illegal mining and the expansion of acai production, have had measurable epidemiological consequences. These activities are consistently associated with increased malaria incidence due to favorable conditions for Anopheles vectors^6^. Moreover, the commercialization of acai has contributed to the emergence of foodborne transmission of Chagas disease^7^.

### Ethics

This study was approved by the institutional Research Ethics Committee (CAAE Nº 53153916.7.0000.0065). All data were anonymized prior to analysis, and access to secondary data from the Laboratory Environment Manager (GAL) and the Notifiable Diseases Information System (SINAN) was restricted to variables necessary for epidemiological characterization (with authorization from health surveillance authorities).

## MATERIAL AND METHODS

### Study design and population

An observational, cross-sectional, retrospective, and descriptive study was conducted using a convenience sample. Serum specimens were collected from patients presenting with AFI during routine diagnostic procedures in public health laboratories. Samples from 727 individuals, collected in 2018 (n = 20), 2019 (n = 581), and 2020 (n = 126), were analyzed.

This study aims to find the potential etiologies of viral and other infections in AFI cases reported from 2018 to 2022 in the Amazon region of Amapa State, Brazil. AFI, with a fever duration cutoff of 2-7 day from onset, was chosen as the inclusion criterion.

### Laboratory procedures

Specimens were initially processed at the Central Public Health Laboratory of Amapa State (LACEN-AP) following routine diagnostic protocols, including arbovirus screening when clinically indicated. Additional tests, such as serology and polymerase chain reaction (PCR) for other pathogens, were performed at the discretion of attending physicians, with results recorded on GAL. Confirmatory local methods included enzyme linked immunosorbent assay (ELISA) for immunoglobulin M (IgM) and NS1 (the latter exclusively for dengue), electrochemiluminescence, reverse transcription PCR (RT-PCR), and sequencing; for bacterial and fungal infections, ELISA for leptospirosis and blood, urine, and stool cultures were performed; whereas parasitic infections were confirmed using thick blood smears, the Strout test, electrochemiluminescence, and immunochromatographic tests.

After initial processing, samples were stored at −20 °C and shipped to the Virology Laboratory of the Institute of Tropical Medicine of Sao Paulo, Faculty of Medicine, University of Sao Paulo (LV-IMT-FMUSP, LIM-52), in which they were preserved at −80 °C until further testing.

### Molecular screening

At LV-IMT-FMUSP, samples underwent real-time PCR assays targeting viral agents. The screening included assays for Pan-Orthoflavivirus and Pan-Alphavirus. The following viral agents were tested:

- Flaviviruses, Alphaviruses, Orthobunyaviruses: yellow fever^8^, dengue, zika, chikungunya, mayaro, and oropouche viruses^9^.
- Genus Orthoflavivirus^10^: Japanese encephalitis, Russian spring-summer encephalitis, tick-borne encephalitis, Usutu, West Nile, and Saint Louis encephalitis viruses.
- Genus Alphavirus^11^: Ross River, O’nyong-nyong, Sindbis, Barmah Forest, Eastern equine encephalitis, Western equine encephalitis, and Venezuelan equine encephalitis viruses.

Additional real-time PCR assays included pathogens with overlapping clinical presentations, such as enterovirus^12^, parvovirus B19^13^, hepatitis E virus (HEV)^14^, and Leptospira spp^15^.

### GAL

In this study, diagnostic test results from LACEN-AP were retrieved via GAL.

GAL, the national computerized system, was designed to manage and monitor laboratory routines within the National Public Health Laboratory Network. It is managed by the Ministry of Health via the General Coordination of Public Health Laboratories and tracks the flow of biological samples from collection to final diagnosis, standardizing results and providing real-time data for the surveillance of high-priority and compulsory notification diseases.

### SINAN

SINAN, the primary national surveillance database managed by the Brazilian Ministry of Health, is a decentralized system fed by the mandatory notification of diseases and conditions in the National Compulsory Notification List.

SINAN provides high-resolution microdata (individual-level records) that include clinical and demographic variables (age, sex, pregnancy status, municipality of residence, reported condition, date of symptom onset, and clinical manifestations), diagnostic test results, among others.

### Integration of data

Clinical and demographic variables (age, sex, municipality of residence, reported condition, date of symptom onset, and clinical manifestations) were combined with GAL laboratory results. Using this integrated dataset, data analysis was strictly descriptive, focusing on characterizing the reported cases.

For the diagnostic tests either at LACEN or IMT, samples were classified as positive if they were reactive in at least one assay.

### Statistical analysis

Continuous variables are shown as medians and interquartile ranges (IQR), and categorical ones, as frequencies. Subgroup pairwise comparisons were performed via the Pearson’s chi-square or Fisher’s exact tests. Correlation between monthly cases and NASA POWER precipitation data was assessed using Spearman’s rank test. The Benjamini-Hochberg procedure was applied to control the false discovery rate across multiple comparisons. Spatial distribution maps were generated using the geobr, sf, and ggplot2 packages. Statistical significance was set at p < 0.05, using R(version 4.4.0) via RStudio.

## RESULTS

### Demographic and clinical characteristics of the population

The study population showed a balanced distribution between biological sexes, with a slight predominance of females, accounting for 50.2% (n = 365) of the cases, whereas males represented 49.2% (n = 362). Regarding age, the female sex presented a median age of 28 years (interquartile range : 15-45), with values ranging from

0.008 to 90 years. For males, the median age was 26 years (IQR: 12-42), ranging from 0.17 to 88 years.

Table 1 comparatively analyzes the signs, symptoms, and baseline comorbidities in patients with confirmed dengue, those positive for other pathogens (non-dengue), and individuals with negative test results.

According to Table 1, 491/727 (67.5%) individuals presented complete clinical information. Fever was followed by headache, myalgia, low back pain, nausea, and vomiting. Other manifestations included rash, retro-orbital pain, arthritis, arthralgia, conjunctivitis, and petechiae, with laboratory findings of leukopenia. Chronic comorbidities included systemic arterial hypertension, diabetes mellitus, and chronic kidney disease. Autoimmune diseases and isolated cases of hematological, hepatic, and peptic disorders also occurred.

**Table 1:** Clinical characteristics in dengue-positive, other pathogen-positive (non-dengue), and negative tests.

Signs, symptoms and comorbidity	Dengue*	Non-dengue	Negative	p-value*
	(n=109)	(n=76)	(n=542)	
Fever	109 (100.0)	76 (100.0)	542 (100.0)	NS
Headache	75 (68.8)	38 (50.0)	291 (53.7)	0.010a,0.004b
Myalgia	63 (57.8)	35 (46.1)	224 (41.3)	0.002b
Back pain	44 (40.4)	24 (31.6)	164 (30.3)	0.039b
Vomiting	38 (34.9)	23 (30.3)	138 (25.5)	0.044b
Nausea	35 (32.1)	28 (36.8)	159 (29.3)	NS
Rash	32 (29.4)	22 (28.9)	81 (14.9)	<0.001b,0.002c
Retro-orbital pain	28 (25.7)	16 (21.1)	85 (15.7)	0.012b
Arthralgia	30 (27.5)	14 (18.4)	76 (14.0)	<0.001b
Arthritis	27 (24.8)	14 (18.4)	81 (14.9)	0.012b
Conjunctivitis	13 (11.9)	7 (9.2)	24 (4.4)	0.002b
Petechiae	6 (5.5)	5 (6.6)	23 (4.2)	NS
Positive tourniquet test	5 (4.6)	0 (0.0)	2 (0.4)	0.002b
Leukopenia	2 (1.8)	2 (2.6)	7 (1.3)	NS
**Comorbidities**				
Hypertension	10 (9.2)	2 (2.6)	27 (5.0)	NS
Autoimmune diseases	2 (1.8)	0 (0.0)	2 (0.4)	NS
Diabetes	1 (0.9)	0 (0.0)	9 (1.7)	NS
Chronic kidney disease	1 (0.9)	2 (2.6)	0 (0.0)	0.015c
Hematological diseases	0 (0.0)	0 (0.0)	1 (0.2)	NS
Hepatopathies	0 (0.0)	0 (0.0)	1 (0.2)	NS
Acid peptic disease	0 (0.0)	0 (0.0)	1 (0.2)	NS
Not informed	23 (21.1)	30 (39.5)	183 (33.8)	0.007a,0.009b

p-values calculated using the chi-squared or Fisher’s exact tests across the three groups. Statistically significant pair: ^a^Dengue vs. Non-dengue (p<0.05); ^b^Dengue vs. Negative (p<0.05); ^c^Non-dengue vs. Negative (<0.05); NS = non-significant; ^*^Dengue cases, including co-infections with other pathogens.

Of the 727 notifications, 585 (80.5%) had arbovirus infection as the initial diagnostic hypothesis—predominantly dengue, followed by chikungunya, zika, and yellow fever. The remaining 142 individuals (19.5%) presented suspicions of other conditions, including Chagas disease, pertussis, COVID-19, diphtheria, Q fever, viral hepatitis, visceral leishmaniasis, leptospirosis, rubella, measles, and toxoplasmosis.

LACEN/AP performed 1,239 diagnostic tests, with 195 positive results (15.7%). Dengue was the most frequent diagnosis: 109 cases out of 650 tests (16.8%). Other notable findings included leptospirosis (20/98; 20.4%), chikungunya (16/86; 18.6%), and measles (15/53; 28.3%).

Remaining positive cases involved zika (9/62; 14.5%), parvovirus B19 (6/21; 28.6%). Other positive tests for other pathogens were bacterial infections (5/17; 29.4%), Chagas disease (1/80; 1.3%), malaria (2/80; 2.5%), CMV (1/33; 3.0%), hepatitis A (2/24; 8.3%); hepatitis B (1/20; 5.0%), COVID-19 (2/4; 50.0%), influenza virus (1/2; 50%), fungal (1/1; 100%), and arbovirus screening (4/8, 50.0%).

At LV-IMTFMUSP, molecular surveillance involved testing 727 individuals by RT-PCR, depending on sample availability. The analysis found positive cases for parvovirus B19 (12/727; 1.66%), enterovirus (2/712; 0.28%), zika virus (1/727; 0.14%), and mayaro virus (1/727; 0.14%). Tests found no viral RNA or bacterial DNA for dengue (0/727; 0%), chikungunya (0/727;0 %), yellow fever (0/709; 0%), hepatitis E (0/703; 0%), or leptospirosis (0/641;0%). All samples tested negative in pan-Orthoflavivirus and pan-Alphavirus screening assays (0/727; 0.0%).

At LV-IMT-FMUSP, PCR analysis of all 727 serum samples found only 16 positive results, four of which confirmed or complemented LACEN/AP findings. These results found 209 diagnoses in 185 individuals across both laboratories (Table 2), including 23 cases of co-infection (Table 3).

Dengue was the most prevalent arbovirus, with 109 confirmed detections (2019: n=82; 2020: n=18; 2018: n=9). Chikungunya virus occurred in 16 cases, mostly in 2019 (n=8). Zika virus accounted for 10 occurrences from 2018 to 2020. Mayaro virus occurred in one confirmed case (2019). Tests found no yellow or oropouche fever. Exanthematous viral infections included 33 detections (parvovirus B19: n=16; measles: n=15; Enterovirus sp.: n=2). Respiratory viral infections comprised three cases (COVID-19: n=2; influenza: n=1). Viral hepatitis included three cases (hepatitis A: n=2; hepatitis B: n=1). Other infections included 26 bacterial or fungal detections, predominantly leptospirosis (n=20), with isolated cases of Staphylococcus aureus, Salmonella typhi, Staphylococcus hominis, Escherichia coli, and Candida glabrata. Parasitic infections comprised three cases (malaria: n=2; Chagas disease: n=1).

### Precipitation influence

Analysis of the temporal relationship showed a moderate positive correlation concurrent with rainfall (lag 0: ρ=0.459, nominal p=0.0416), which lost statistical significance after the Benjamini-Hochberg correction. Figure 1 shows the monthly distribution of dengue cases and precipitation in Amapa State in the study period. The distribution of the other diagnosed pathogens had a sample size too small to observe any correlation with precipitation.

### Distribution of positive cases per region

The spatial distribution maps show distinct epidemiological patterns across Amapa State, Brazil, with southeastern municipalities consistently enduring the highest disease burden across nearly all conditions (Figure 2). Among the arboviral diseases, dengue emerged as the most widespread and severe, totaling up to 50 cases per municipality and primarily impacting the southeastern and central-western regions. In contrast, zika and chikungunya showed substantially lower case counts and more restricted geographic distributions; zika appeared sparsely in the northern and southeastern areas, whereas chikungunya mostly occurred in the northernmost and southeastern coastal municipalities.

**Table 2:** Test results across both laboratories according to method.

Disease	Reactive tests (n)	Methods (n)	Origin
Dengue	109	ELISA IgM (108); RDT IgM (1)	LACEN
Lepstospirosis	20	ELISA IgM (20); RDT IgM (1)	LACEN
Chikungunya	16	ELISA IgM (15); RDT IgM (1)	LACEN
Parvovirus B19	16	ELISA IgM (6);	LACEN LV-IMT
		PCR (10)	
Measles	15	ELISA IgM (15); RT-PCR (4); Sequencing (2)	LACEN
Zika	10	ELISA IgM (4); RDT IgM (5); RT-PCR (1)	LACEN LV-IMT
Bacterial infection	5	Blood culture (3); Urine culture (1); Stool culture (1)	LACEN
Arbovirus screening	4	HI (4)	LACEN
Hepatitis A	2	Electrochemiluminescence IgM (2)	LACEN
Malaria	2	TBS (2)	LACEN
COVID-19	2	RT-PCR (2)	LACEN
Enterovirus	2	RT-PCR (2)	LV-IMT
Cytomegalovirus	1	Electrochemiluminescence IgM (1)	LACEN
Mayaro	1	RT-PCR (1)	LV-IMT
Hepatitis B	1	Electrochemiluminescence HBsAg e Anti-HBe (1)	LACEN
Influenza	1	RT-PCR (1)	LACEN
Fungal infection	1	Urine culture (1)	LACEN
Chagas disease	1	TBS e Strout (1)	LACEN
TOTAL	209	-	-

ELISA = Enzyme-linked immunosorbent assay; RDT = Rapid diagnostic test; MAT = Microscopic agglutination test; HI = Hemagglutination inhibition; RT-PCR = Reverse transcription polymerase chain reaction; TBS = Thick blood smear.

**Table 3:** Distribution of cases of co-infection according to test results and methods across laboratories.

Disease	Cases (n)	Methods (n)	Origin
Dengue + Measles	7	ELISA Dengue IgM (7); ELISA Measles IgM (7)	LACEN
Dengue + Zika	2	ELISA Dengue IgM (2); ELISA Zika IgM (1); RDT Zika IgM (1)	LACEN
Dengue + Chikungunya	2	ELISA Dengue (2); ELISA Chikungunya IgM (2)	LACEN
Dengue + Parvovirus B19	2	ELISA Dengue IgM (2); ELISA Parvovirus IgM (1) PCR Parvovirus B19 (1)	LACEN LV-IMT
Dengue + Chagas disease	1	ELISA Dengue IgM (1); TBS (1)	LACEN
Dengue + Hepatitis A	1	ELISA Dengue IgM (1); Electrochemiluminescence Anti-HAV IgM (1)	LACEN
Dengue + *Escherichia coli*	1	ELISA Dengue IgM (1); *E. coli* (1)	LACEN
Chikungunya + Parvovirus B19	1	ELISA Chikungunya IgM (1) PCR Parvovirus B19 (1)	LACEN LV-IMT
Chikungunya + Malaria	1	ELISA Chikungunya IgM (1); TBS (1)	LACEN
Leptospirosis + *Salmonella typhi*	1	ELISA Leptospirosis IgM (1); Stool culture (1)	LACEN
Leptospirosis + *Staphylococcus hominis*	1	ELISA Leptospirosis IgM (1); Blood culture (1)	LACEN
Hepatitis A + *Candida glabrata*	1	Electrochemiluminescence Anti-HAV IgM (1); Urine culture (1)	LACEN
Zika + Measles	1	ELISA Zika IgM (1); ELISA Measles IgM (1)	LACEN
Dengue + Parvovirus B19 + Measles	1	ELISA Dengue IgM (1); ELISA Parvovirus B19 IgM (1); ELISA Measles IgM (1) and RT-PCR Measles (1)	LACEN
TOTAL	23	-	-

ELISA = Enzyme-linked immunosorbent assay; RDT = Rapid diagnostic test; RT-PCR = Reverse transcription polymerase chain reaction; TBS = Thick blood smear.

**Figure 1:**
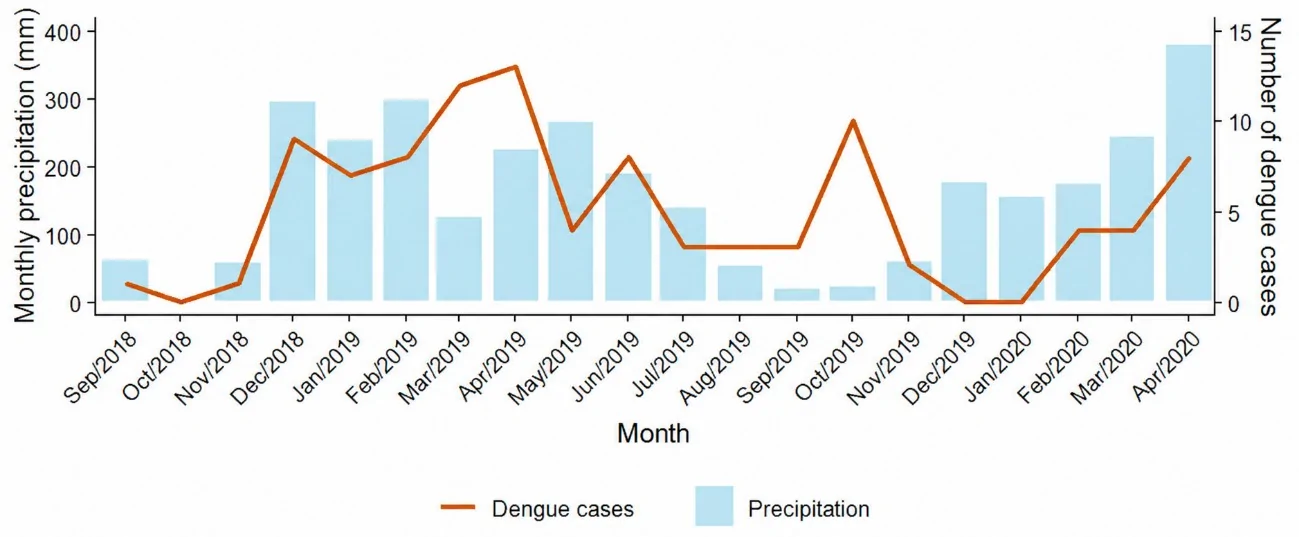
Monthly distribution of dengue cases and precipitation in Amapa State, Brazil, from September 2018 to April 2020. Precipitation represents the monthly mean across the 16 municipalities in Amapa State.

Measles, parvovirus B19, and leptospirosis showed a nearly identical hotspot pattern: sharply concentrated within the same southeastern cluster, although their overall volumes were significantly lower, peaking at 10 cases. Within this group, parvovirus B19 showed the largest reach, with low-level case counts spreading into the northern and western parts of the state, whereas measles and leptospirosis remained tightly confined to the southeastern coast.

## DISCUSSION

This study investigated the etiologies of AFI cases reported in Amapa State from 2018 to 2022. Of 727 evaluated individuals, definitive diagnoses established 185 cases (25.4%), including 23 cases of co-infection.

Our findings are in line with previous regional studies. Dengue remains the predominant AFI etiological agent in Latin America, with chikungunya and zika showing an increased prevalence. Leptospirosis, though often overlooked, represents a prevalent bacterial contributor^1^,^16^.

**Figure 2:**
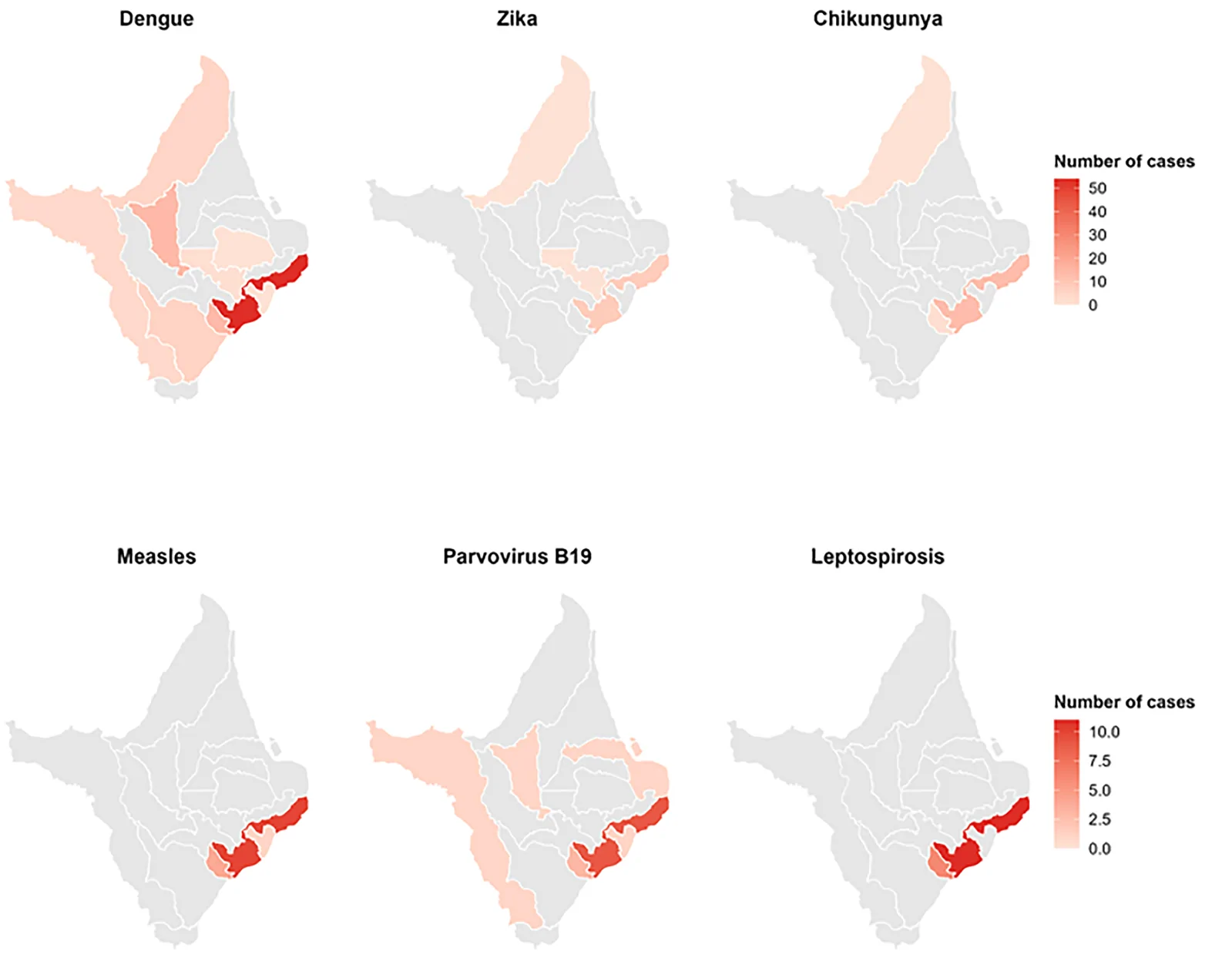
Spatial distribution of arboviral and other disease cases by municipality in Amapa State, Brazil.

Notably, this study found one case of mayaro virus infection, consistent with its rare occurrence in Amapa State (six confirmed cases nationwide in 2015-2025)^17^. Following systematic PCR testing, no individual in our cohort was positive for Oropouche virus disease, despite epidemiological data (2015-2025) reporting 16,571 cases nationwide, including 218 in Amapa State. A recent increase in its detection (2023-2024) likely reflects greater diagnostic capacity and active case-finding^17^. These results suggest Amapa State may represent a zone of “silent expansion” for Oropouche virus disease, in which overlapping arbovirus transmission obscures true disease burden. Strengthening surveillance, expanding laboratory capacity, and prioritizing differential diagnostics are essential to capture epidemiological dynamics and guide interventions. While multiplex syndromic panels are ideal, widespread molecular testing faces strict budget and logistical constraints within the public health system in Amapa State. A more sustainable solution could be a tiered “reflex testing” cascade at LACEN-AP. Under this protocol, a negative molecular result for primary arboviruses (dengue/zika/chikungunya) would automatically trigger reflex testing for leptospirosis and parvovirus B19 using the original biological sample. This flow could optimize laboratory resources, cut costs, and bypass initial clinical triage blind spots without requiring secondary patient appointments. Lessons from the Amazon highlight the global importance of integrated arbovirus surveillance in regions with overlapping transmission.

The fact that 80.5% of initial hypotheses were generically classified as suspected arboviruses exposes a profound “dengue-centric” bias. Because the early symptoms of dengue, parvovirus B19, and leptospirosis overlap almost entirely, physical triage alone is insufficient. To address this, the regional health system must implement targeted continuing medical education focusing on broad syndromic classifications—such as “acute exanthematous febrile syndrome”—rather than single-pathogen guessing. Furthermore, standard patient intake forms should mandate structured epidemiological risk questions regarding recent floodwater exposure (leptospirosis), mining or forest exposure (malaria/arboviruses), and artisanal acai consumption (acute Chagas disease) to guide clinical suspicion before laboratory results are available.

Leptospirosis remains a critical public health challenge in Amapa State linked to seasonal flooding, precarious sanitation, and transmission in wetland and stilt-house communities^18^ ^,^ ^19^. Despite its prevalence, underreporting persists due to symptomatic overlap with malaria and dengue^20^. Our finding that Leptospira spp. was the second most prevalent confirmed pathogen underscores its critical standing in the regional etiology of AFI. Unlike arboviral infections, which only require supportive care, leptospirosis demands timely antibiotic therapy to prevent severe, potentially fatal complications such as Weil’s disease or pulmonary hemorrhage syndrome. Relying entirely on early clinical differentiation or waiting days for laboratory confirmation in highly endemic zones often results in catastrophic therapeutic delays. Therefore, in areas with high dengue and leptospirosis co-endemicity, clinical protocols must explicitly support the initiation of empirical antibiotic treatment (such as doxycycline or amoxicillin) for patients presenting with severe febrile syndromes and clear epidemiological risk factors, prioritizing clinical survival over definitive laboratory timelines.

Parvovirus B19 occurred in 16 cases (7.6%), representing a notable proportion of AFI diagnoses. Its clinical recognition remains challenging as its early symptoms closely mimic arboviruses such as dengue, Zika, and chikungunya. In tropical regions, B19V is often misdiagnosed as dengue due to overlapping presentations of fever, headache, and rash^21^ ^,^ ^22^. The post-pandemic resurgence of B19V in 2024-2025 marked a significant epidemiological shift driven by an “immunity gap” in pediatric populations. IgM positivity peaked at ~40% in children aged 5-9 years and ~10% in adults, complicating AFI differential diagnosis^23^.

Of the 727 investigated individuals, viral hepatitis was suspected in 10 cases (1.4%), with three confirmed cases of hepatitis A and B (1.4%). No HEV cases were identified despite evidence that HEV genotype 3 is a zoonotic pathogen of concern in Brazil^24^ ^,^ ^25^.

Meta-analyses estimate anti-HEV IgG seroprevalence at ~6% nationally, with regional variations: ~3% in the Amazon and up to 14.2% in rural Rondônia^26^ ^-^ ^28^. Diagnostic invisibility remains a major challenge as public health screening routinely ignores HEV testing, particularly in vulnerable Amazonian populations. Overlapping clinical presentations with hepatitis b and d further obscure HEV detection, suggesting that some acute hepatitis cases of “unknown etiology” may represent undiagnosed HEV.

Measles occurred in 15 cases (7.1%). Despite mass vaccination programs, Brazil lost its measles-free status in 2018 following outbreaks linked to Venezuelan migration and low vaccination coverage^29^ ^,^ ^30^. Although elimination status was regained in 2023-2024, new cases in Tocantins in 2025 highlight ongoing vulnerability. The porous border of Amapa State with French Guiana and high mobility of illegal gold miners create a unique epidemiological corridor sustaining measles and other infectious diseases.

Malaria transmission remains a major concern in Oiapoque, one of the highest transmission zones in the Americas. Despite persistent “silent” transmission by asymptomatic carriers in mobile populations, our cohort only included two malaria cases (0.95%), reflecting the predominance of samples collected in Macapa. Epidemiological data shows a downward trend in Amapa State, with a 16.8% reduction in cases from 2024 to 2025 and an 82% decline in Macapa. Nonetheless, the Oiapoque border remains the main epidemiological challenge in the state^31^ ^-^ ^34^. The cohort included a case of acute Chagas disease, reflecting the specific epidemiological profile of Amapa State. From 2018 to 2022, the state remained a high-endemicity region for acute Chagas disease in the Brazilian Amazon, with oral transmission—primarily by consumption of artisanal acai and bacaba juice—emerging as the dominant route of infection^35^ ^,^ ^36^.

A notable finding in this study was the elevated detection of multiple infectious agents. Across 727 patients, this study found 209 reactive results, with co-detection of distinct pathogens in 23 cases. Similar patterns have been reported in regions endemic for malaria and arboviruses, in which simultaneous infections are confirmed by multiple molecular assays ^37^ ^,^ ^38^. In such contexts, potential cross-reactivity further compounds diagnostic complexity. The cross-reactivity between zika, dengue, and yellow fever serological assays, for example, is primarily attributed to the high degree of structural homology within the envelope protein in members of the Orthoflavivirus genus^39^.

The seasonal transmission pattern in this study likely reflects the equatorial climate of the region, in which persistently high precipitation and elevated temperatures sustain vector breeding sites throughout the year. Thus, the continuous circulation of febrile illnesses in Amapa State may obscure the more distinct seasonal peaks and lag patterns in other climatic settings. Because these vectors are ectothermic, sustained warmth and humidity likely support ongoing reproduction and pathogen transmission, potentially limiting the applicability of traditional rainfall-based predictive models^40^.

Furthermore, the geographic distribution of cases across the state shows a pronounced clustering pattern in the southeastern municipalities. This phenomenon shows how urban border environments create convergent risk factors for diseases with entirely different transmission mechanisms. Consequently, these findings highlight the critical importance of integrated urban health interventions that can simultaneously address environmental and social determinants of health.

### Strengths and limitations

This study offers several strengths. The use of a well-defined dataset allowed for systematic analyses and ensured consistency in the examined variables. The use of a convenience sample with a retrospective design enabled the inclusion of a large sample size. Moreover, its reliance on real-world data increases the relevance of the findings for clinical and policy contexts.

Nonetheless, certain limitations deserve acknowledgement. First, as a retrospective analysis based on secondary data, this study is subject to the inherent constraints of such designs, particularly the possibility of selection bias. Second, the reliance on existing records may have introduced inaccuracies or missing information, which could affect the validity of its findings: specifically, high rates of missing or inconsistent dates in GAL forms prevented the reliable calculation of the average time from symptom onset to sample collection. This is a critical factor given the reliance of our algorithm on RT-PCR for early acute samples (up to seven days) and IgM for longer intervals, meaning that geographical barriers and delayed healthcare access in remote areas of Amapa State may have caused some patients to miss the acute window for molecular detection. Third, because this study involved a specific population and context, the generalizability of its results to other settings may be restricted. Finally, the conservation of biological samples represents a potential source of bias. Inadequate storage or handling conditions can compromise nucleic acid integrity, and for molecular assays such as PCR, this may reduce sensitivity or generate false negatives, thereby distorting diagnostic accuracy.

## CONCLUSIONS

The causes of AFI in Amapa are multiple and may involve infection by more than one etiological agent. This underscores the complexity of diagnostic accuracy in regions with overlapping multiple arbovirus and bacterial transmission. The detection of rare pathogens such as Mayaro virus and acute Chagas disease reflects the unique ecological and epidemiological profile of the region. These findings reinforce the role of Amapa State as a sentinel for infectious disease surveillance as its porous borders, ecological connectivity, and high human mobility sustain transmission corridors. Strengthening laboratory capacity, integrating differential diagnostics, and expanding surveillance systems are essential to capture the true burden of AFI and to guide targeted interventions. While acknowledging the limitations inherent to retrospective designs and diagnostic constraints, this study emphasizes the global relevance of integrated surveillance in ecologically dynamic regions. Crucially, addressing these challenges requires improving data completeness, such as by the meticulous entry and digital optimization of GAL forms, to ensure accurate epidemiological tracking in complex equatorial settings.

## Data Availability

The complete anonymized dataset supporting the findings of this study is included in this article.
